# Rapid identification of tomato Sw-5 resistance-breaking isolates of *Tomato spotted wilt virus* using high resolution melting and TaqMan SNP Genotyping assays as allelic discrimination techniques

**DOI:** 10.1371/journal.pone.0196738

**Published:** 2018-04-30

**Authors:** Valentina di Rienzo, Giovanni Bubici, Cinzia Montemurro, Fabrizio Cillo

**Affiliations:** 1 Spin off SINAGRI s.r.l., Bari, Italy; 2 Istituto per la Protezione Sostenibile delle Piante, Consiglio Nazionale delle Ricerche, Bari, Italy; 3 Dipartimento di Scienze del Suolo, della Pianta e degli Alimenti, Università degli Studi di Bari "Aldo Moro", Bari, Italy; University of New England, AUSTRALIA

## Abstract

In tomato, resistance to *Tomato spotted wilt virus* (TSWV) is conferred by the dominant gene, designated Sw-5. Virulent Sw-5 resistance breaking (SRB) mutants of TSWV have been reported on Sw-5 tomato cultivars. Two different PCR-based allelic discrimination techniques, namely Custom TaqMan™ SNP Genotyping and high-resolution melting (HRM) assays, were developed and compared for their ability to distinguish between avirulent (Sw-5 non-infecting, SNI) and SRB biotypes. TaqMan assays proved to be more sensitive (threshold of detection in a range of 50–70 TSWV RNA copies) and more reliable than HRM, assigning 25 TSWV isolates to their correct genotype with an accuracy of 100%. Moreover, the TaqMan SNP assays were further improved developing a rapid and simple protocol that included crude leaf extraction for RNA template preparations. On the other hand, HRM assays showed higher levels of sensitivity than TaqMan when used to co-detect both biotypes in different artificial mixtures. These diagnostic assays contributed to gain preliminary information on the epidemiology of TSWV isolates in open field conditions. In fact, the presented data suggest that SRB isolates are present as stable populations established year round, persisting on both winter (globe artichoke) and summer (tomato) crops, in the same cultivated areas of Southern Italy.

## Introduction

*Tomato spotted wilt virus* (TSWV) [1] is the type member of the plant-infecting genus *Tospovirus* in the family *Bunyaviridae* [2]. The TSWV genome consists of three negative-sense or ambisense RNA segments denoted as L, M and S. Segment L is a negative sense RNA that encodes the viral component of the RNA dependent RNA polymerase [3]. Segment M is an ambisense RNA encoding the cell-to-cell movement protein, NSm [4], and the precursor of the surface glycoproteins, Gn and Gc, involved in TSWV transmission by thrips [5, 6]. Segment S, an ambisense RNA as well, encodes a silencing suppressor, NSs [7], and the nucleocapsid protein, N [8].

TSWV is efficiently vectored by several species of thrips (*Thysanoptera*: *Thripidae*), including *Frankliniella occidentalis*, in a persistent and propagative manner [9]. A century after the ‘spotted wilt’ disease of tomato first occurred in Australia [10], TSWV is still a major agricultural threat, with a severe impact on many food and ornamental crops (>1000 species), causing crop disease epidemics of worldwide economic significance [11, 12]. For this reason, TSWV is included in the OEPP/EPPO A2 list of pests recommended for regulation as a quarantine pathogen, since not widely distributed throughout Europe [13]. Information about the presence, occurrence, and genetic diversity of TSWV in many areas is limited, although such data on a devastating viral pathogen subjected to quarantine measures are pivotal for disease management.

The control of TSWV-induced diseases in crop plants is a problematic challenge because of the wide range of host plant species and the difficulties in deploying efficient control measures against thrip vectors [14]. Consequently, an intense research work was directed to develop long-term strategies based on plants genetically resistant against TSWV. A single dominant resistance (R) gene, named Sw-5, was found responsible of the inheritance of a useful vertical resistance [15]. Sw-5 was characterised as a coiled-coil/nucleotide-binding site/leucine-rich-repeats (CC-NBS-LRR) protein belonging to the class 2 of tomato R genes [16]. However, since then, Sw-5 resistance-breaking (SRB) isolates emerged in several countries worldwide, including Italy and Spain, Australia, South Africa, Hawaii and recently also California [17–24]. In fact, TSWV is characterised by a high genetic variability and mutability, determined by the diversity among isolates diffused and persistent year-long in a broad host range, by the replication within its thrips vector and by the ability to exchange the segments of its tripartite genome between different isolates [25]. Therefore, it is not surprising that as soon as Sw-5 mediated resistance was deployed worldwide in commercial tomato hybrids, resistance breaking TSWV isolates started to be reported. It has been suggested that SRB isolates, under a high selective pressure due to the extensive use of resistant varieties in the field, could rapidly become the predominant field isolates in tomato growing areas [26]. In a glasshouse experiment, SRB isolates emerged on 3% of the resistant tomato plants, and after four successive passages on resistant varieties were shown to be very stable, competitive in mixed infections with the original avirulent isolates (Sw-5 non-infecting or SNI) and always able to display virulent infection on Sw-5 resistant genotypes [23]. In Spanish populations of TSWV isolates, the ability to overcome Sw-5 resistance was associated to either of two mutations in the RNA M nucleotide sequence, leading to two amino acid changes at positions 118 (C118Y) or 120 (T120N) of the NSm protein, respectively [27]. However, the former single nucleotide polymorphism (SNP) was largely predominant in Spain, and was the only detected by nucleotide sequencing of viral cDNA from SRB TSWV isolates in California [17].

Quick and reliable detection of SNP variants in TSWV isolates is required for identifying SRB population distribution and extent, and deploying appropriate control measures. To avoid cloning and sequencing procedures, innovative technologies based on polymerase chain reaction (PCR) have been used for genotyping. Real-time PCR based on TaqMan™ fluorescent probes has been proposed as an allelic discrimination technique, able to detect SNP variants in populations of RNA viruses such as TSWV, *Potato virus Y* (PVY) and *Beet necrotic yellow vein virus* (BNYVV) [28–30]. In addition, another real-time PCR-based SNP identification technique, the high resolution melting (HRM) DNA curve analysis [31], provides an alternative rapid approach to the use of probes and direct DNA sequencing, but has been seldom proposed as a method for plant virus and viroid genotyping [32–34].

Aim of the present study is to test and compare two different allelic discrimination techniques, namely Custom TaqMan™ SNP genotyping and HRM assays, for their ability to distinguish accurately between SNI and SRB isolates in TSWV open field populations. Moreover, the present work aims to develop a innovative tool information about the current presence of the SRB TSWV biotype in Apulia region in Southern Italy, a very important processing tomato growing area heavily affected by the spotted wilt disease, thus contributing to fundamental epidemiological knowledge.

## Results

### Collection of TSWV isolates

Samples of tomato and artichoke were collected in summer and in winter seasons, respectively. Leaves were harvested from field crops showing viral symptoms at different locations in Southern Italy, where TSWV outbreaks have occurred on Sw-5-carrying tomato hybrids in recent years. From a selected number of field samples, the cDNA fragment corresponding to the viral NSm gene was RT-PCR amplified and sequenced to test consistency between sequence- and Real Time PCR-based methods (i.e. HRM and TaqMan probes). Only one SNP responsible for the resistance-breaking phenotype was detected on the NSm gene sequence of SRB isolates, leading to the amino acid change at positions 118 (C118Y). Sw-5 resistant tomato hybrids bore infections by SRB isolates exclusively, whereas non-Sw-5 tomato hybrids and artichoke hosted infections of both biotypes, i.e. the genotypes coding for both the C118 and the Y118 amino acids were detected (Table 1).

**Table 1:** List of isolate name, plant host, and SNP discrimination by sequencing, HRM and Taqman-SNP Genotyping assay.

		SNP Genotyping *				
		Sanger Sequencing	HRM		TaqMan	
ISOLATE	PLANT HOST †		*random primer*	*gene-specific*	*random primer*	*gene-specific*
Borgo 1	Tomato–Sw-5	SRB	SRB	SRB	SRB	SRB
Borgo 2.1	Tomato—Sw-5	SRB	SRB	SRB	SRB	SRB
Borgo 2.2	Tomato—Sw-5	SRB	other	SRB	SRB	SRB
Borgo 2.3	Tomato—Sw-5	SRB	n.a.	SRB	SRB	SRB
T-J	Tomato—s	SNI	n.a.	SNI	n.a.	SNI
15.9	Tomato—s	SNI	n.a.	SNI	*SRB*	SNI
14.28	Artichoke	SNI	SNI	*SRB*	SNI	SNI
L1-1	Artichoke	SNI	SNI	SNI	SNI	SNI
L1-2	Artichoke	SRB	other	other	SRB	SRB
L1-4	Artichoke	SNI	SNI	SNI	SNI	SNI
L1-6	Artichoke	SNI	SNI	SNI	SNI	SNI
L1-7	Artichoke	SNI	SNI	SNI	SNI	SNI
L2-1	Artichoke	SRB	other	SRB	n.a.	SRB
L2-3	Artichoke	SRB	n.a.	SRB	SRB	SRB
L2-4	Artichoke	SNI	SNI	SNI	SNI	SNI
L4-1	Artichoke	SNI	SNI	SNI	SNI	SNI
L4-2	Artichoke	SNI	SNI	SNI	SNI	SNI
L4-4	Artichoke	SNI	SNI	SNI	SNI	SNI
Q1	Tomato—s	SRB	-	SRB	-	SRB
Q2	Tomato—s	n.s.	-	SRB	-	SRB
Q3	Tomato—s	n.s.	-	SRB	-	SRB
Q4-T4	Tomato—s	SNI	-	SNI	-	SNI
Q5	Tomato—s	n.s.	-	SRB	-	SRB
Q6	Tomato—s	n.s.	-	SRB	-	SRB
Q7	Tomato–Sw-5	n.s.	-	SRB	-	SRB
ASSAY SENSITIVITY (%) ‡			77.7	100.0	88.8	100.0
ASSAY ACCURACY (%) ‡			78.6	90.0	93.8	100.0
Error Rate ‡			3/11	2/20	1/16	0/20

*SRB: sequence TGT at codon 118, coding for Cystein (118C); SNI: sequence TAT at codon 118, coding for Tyrosin (118Y); n.a.: failure to amplify; n.s.: not sequenced; other: assigned to cluster other than SNI and SRB; *italics*: misclustering;—: not analysed

†Tomato–Sw-5: Sw-5 tomato genotype; Tomato—s: susceptible, non Sw-5 tomato genotype

‡Assay Sensitivity = number of genotypes obtained/total reactions (%); Assay Accuracy = number of correctly typed samples assuming Sanger sequencing as reference/total reactions (%); Error rate: number of incorrect genotypes/successful amplification assuming Sanger sequencing as reference.

### Validation of HRM and custom TaqMan SNP Genotyping assays for discriminating TSWV isolates

Preliminarily, for standardising the copy number of TSWV M RNA used as the template in HRM and TaqMan assays, equal amounts of the target cDNA were prepared in order to obtain a similar fluorescent signal. A standard curve was built in real-time PCR with the gel-purified amplicon corresponding to nucleotides 426–480 in the TSWV M RNA sequence, using 10-fold dilution series, containing 10^4^−10^1^ target copies. The standard curve covered a dynamic range of four log units of concentrations with R^2^ of 0.991, a slope of –3.129 and an amplification efficiency of 108.7% (not shown). The two samples, named Borgo 1 and 15.9 (Table 2), were chosen as the TSWV SRB and SNI reference panel samples. The absolute quantification of SRB and SNI enabled the detection of as little as 5.86 x 10^1^ and 7.56 x 10^1^ copies, respectively (Table 2).

**Table 2:** Discriminative detection of SNI and SRB by HRM and TaqMan SNP assays in single and artificial mixed samples at different dilutions.

MIX	SNI (copy number)	SRB (copy number)	HRM	TaqMan
15.9	7.56 x 10^5^	-	SNI	SNI
	7.56 x 10^3^	-	No amplification	SNI
	7.56 x 10	-	No amplification	SNI
Borgo 1	-	5.86 x 10^5^	SRB	SRB
	-	5.86 x 10^3^	No amplification	SRB
	-	5.86 x 10	No amplification	SRB
Mix 1	7.56 x 10^5^	5.86 x 10^5^	SNI/SRB	SNI/SRB
Mix 2	7.56 x 10^5^	5.86 x 10^3^	SNI/SRB	SNI
Mix 3	7.56 x 10^5^	5.86 x 10	SNI	SNI
Mix 4	7.56 x 10^3^	5.86 x 10^5^	SNI/SRB	SRB
Mix 5	7.56 x 10^3^	5.86 x 10^3^	SNI/SRB	SNI/SRB
Mix 6	7.56 x 10^3^	5.86 x 10	SNI	SNI
Mix 7	7.56 x 10	5.86 x 10^5^	SRB	SRB
Mix 8	7.56 x 10	5.86 x 10^3^	SRB	SRB
Mix 9	7.56 x 10	5.86 x 10	No amplification	No amplification

A three-step optimization was crucial for attaining reliable HRM-based discrimination of the two alleles. Firstly, since HRM analysis output has been reported to be dependent on the initial amount of PCR template [35], the quality and the concentration of starting cDNA was made equal between the two reference samples, as above described. Secondly, a total of three primer pairs were tested for the ability to identify and distinguish between SRB and SNI isolated from tomato unambiguously (data not shown). Thirdly, an optimization of the amplification temperature conditions was done, testing a touchdown PCR thermal cycle protocol with three different annealing temperature 57, 60 and 63°C and the thermal cycle program suggested by the manufacture’s protocol.

**Fig 1:**
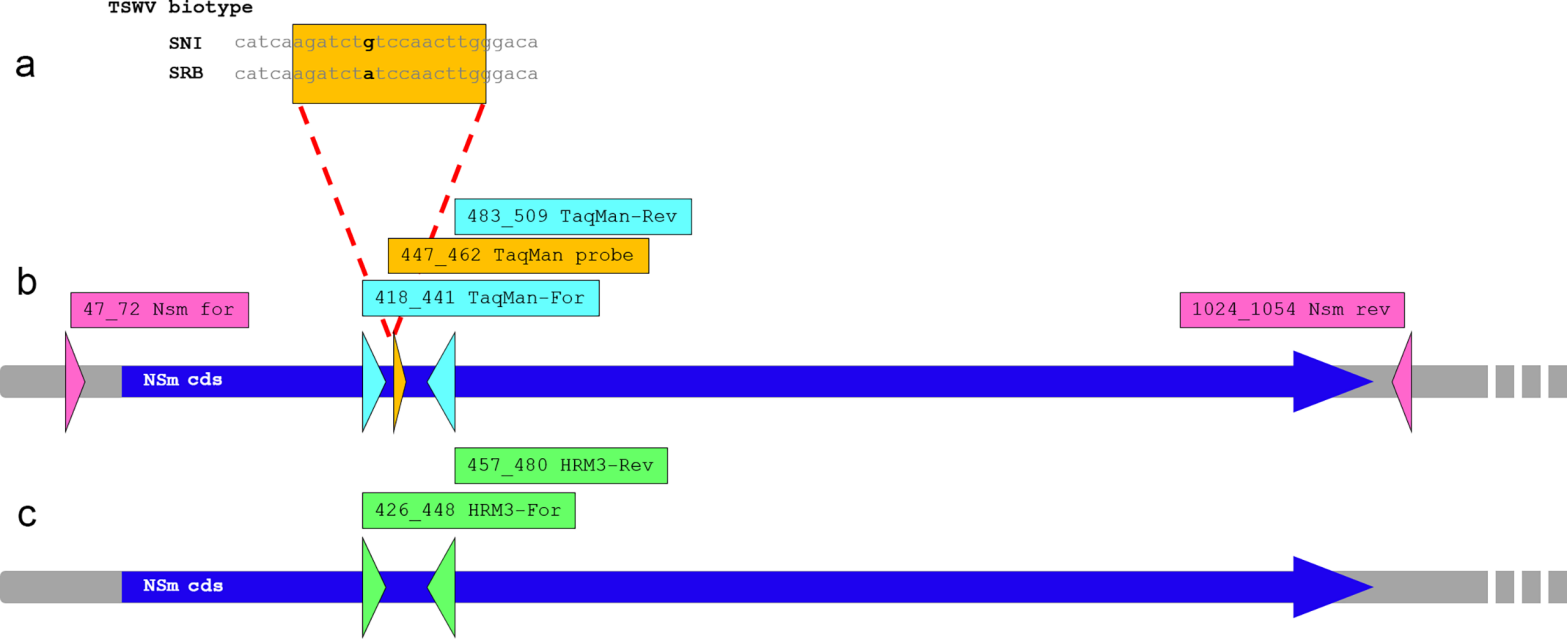
Schematic representation of TSWV NSm gene sequence showing primers and probes localization. (a) the single nucleotide polymorphism (in bold) determining the different pathogenic properties of the SNI and SRB TSWV biotypes. Boxed in orange, the sequence segment chosen for TaqMan probe hybridization. (b) Position of primers (in light blue) and probes (in orange) used in the Custom TaqMan™ SNP Genotyping Assays. In pink, the primer pair used for reverse-transcription-PCR and NSm gene sequencing. (c) Position of primers (green) used in the high-resolution melting assays.

**Fig 2:**
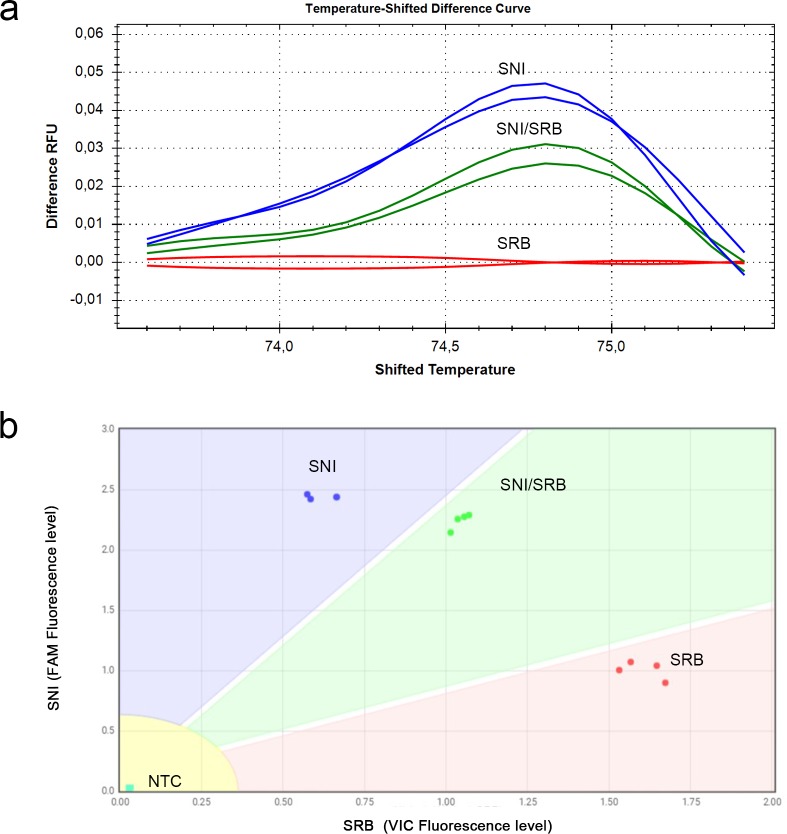
High resolution melting and custom TaqMan SNP Genotyping assays for the detection and discrimination of Sw-5 non infecting (SNI) and Sw-5 resistant breaking (SRB) alleles. (a) The temperature-shifted difference curves discriminated the two reference samples and their mixture in three separated clusters. The two lines correspond to two technical replicates for each sample. (b) Scatter plot of fluorescence data from Custom TaqMan SNP Genotyping assays. Each dot denotes individual replicates (four technical replicates per sample) and corresponds to FAM and VIC fluorescence levels associated to SNI- and SRB-specific probes, respectively. Allele-specific boundaries defined four non-overlapping areas corresponding to SNI, SRB, SNI/SRB mix and no template control (NTC).

The primer pair HRM-3-For and HRM-3-Rev (Fig 1) used in a touchdown protocol at 60°C as the starting annealing temperature, gave the most robust and reproducible assay results, providing a strong fluorescence signal. Three allele-specific melt curves, corresponding to the reference panel samples SRB and SNI and the artificial mixture (SRB/SNI), were obtained with high percent confidence (>90.5%) (Fig 2A).

In parallel, the effectiveness of the TaqMan SNP assay was evaluated (Fig 1). The set-up of the assay was performed with four technical replicates for each sample, including a no template control (NTC). The deriving graphic representation, where the Y and the X axis showed normalized FAM fluorescence intensity (associated to the SNI probe) and VIC fluorescence intensity (associated to the SRB probe), respectively, illustrated the distinction of four areas corresponding to each type of tested samples (Fig 2B). For the NTC samples, the signal corresponded to the background fluorescent level produced by the uncleaved probes. For the sample tested, the fluorescent signal associated with the specific probe was significantly higher than the alternative probe. In particular, NSm_SNI_FAM probe yielded a FAM/VIC signal ratio of about 4, whereas NSm_SRB_VIC probe generated 2 to 3 VIC/FAM signal ratio, in SNI and SRB reference samples respectively (Fig 2B). These results demonstrated the specific hybridisation of the probes to their targets and the absence of significant non-specific signals. When the SNI/SRB mixed sample was amplified, both FAM and VIC fluorescent signals increased significantly when compared to NTC data, reflecting the binding and the cleavage of the two probes during the PCR reaction.

### Discrimination of SNI and SRB mixed samples with HRM and TaqMan SNP assays and their sensitivity

In order to monitor the diffusion of the two genotypes in the field, even co-infecting the same plants, experimental mixtures of SNI and SRB were evaluated by the two assays (Table 2).

**Fig 3:**
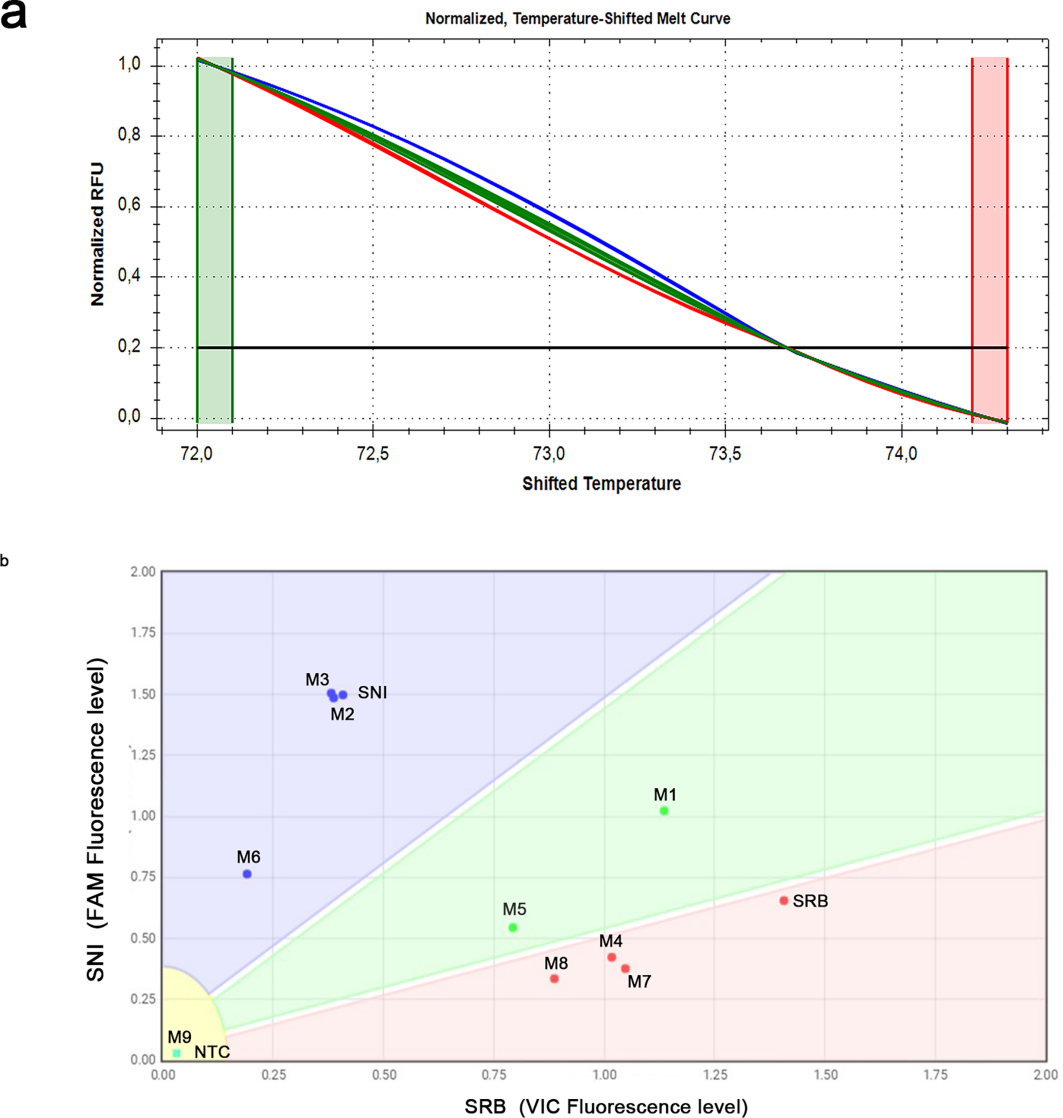
Co-detection and discrimination of SNI and SRB alleles in artificial mixtures by high resolution melting and custom TaqMan SNP Genotyping assays. (a) The normalized temperature-shifted melt curves grouped the artificial mixtures in three clusters: blue, SNI allele (mixtures 3 and 6); green, SNI/SRB alleles (mixtures 1, 2, 4 and 5); red, SRB allele (mixtures 7 and 8). (b) The scatter plot from Custom TaqMan SNP Genotyping assays. SNI and SRB (samples 15.9 and Borgo 1, respectively) were included as the the reference panel samples. Only mixtures 1 and 5 fell within the SNI/SRB boundary. Mixture 9 did not provide amplification signal in both assays.

Success in discriminating mixed samples depended on both the quantity of starting target copy number and the of SNI/SRB ratio. In fact Mix 9, prepared with the lowest copy numbers for both isolates (7.56 x 10^1^ and 5.86 x 10^1^ copy numbers of SNI and SRB, respectively), led to no amplification signals. In the presence of lower copy numbers of either genotypes in unbalanced mixtures (Mix 3, 6, 7, 8), both HRM and TaqMan assays identified only the most abundant genotype, thus fixing the limit for revealing both alleles at 5.86 x 10^3^ and 7.56 x 10^3^ copies of SRB and SNI, respectively. Although samples 1 and 5 were correctly identified as mixtures by both methods, only HRM was able to co-detect both alleles in mixtures 2 and 4 (Fig 3 and Table 2). Therefore, the TaqMan assays showed optimal performances for detecting mixed samples in 1:1 allelic ratio, whereas HRM assay was proven to reveal mixtures containing unbalanced allelic ratios with higher sensitivity.

### HRM and TaqMan SNP assays discriminate SNI and SRB TSWV isolates collected from tomato and artichoke in the field

After the confirmation that single isolates can be efficiently identified by both HRM and Custom TaqMan SNP assays, we evaluated the effectiveness of both approaches on 18 TSWV isolates collected in the field. RNA was extracted from tomato and artichoke infected leaf samples, reverse-transcribed with both random and gene-specific primers and used as the templates for both techniques.

**Fig 4:**
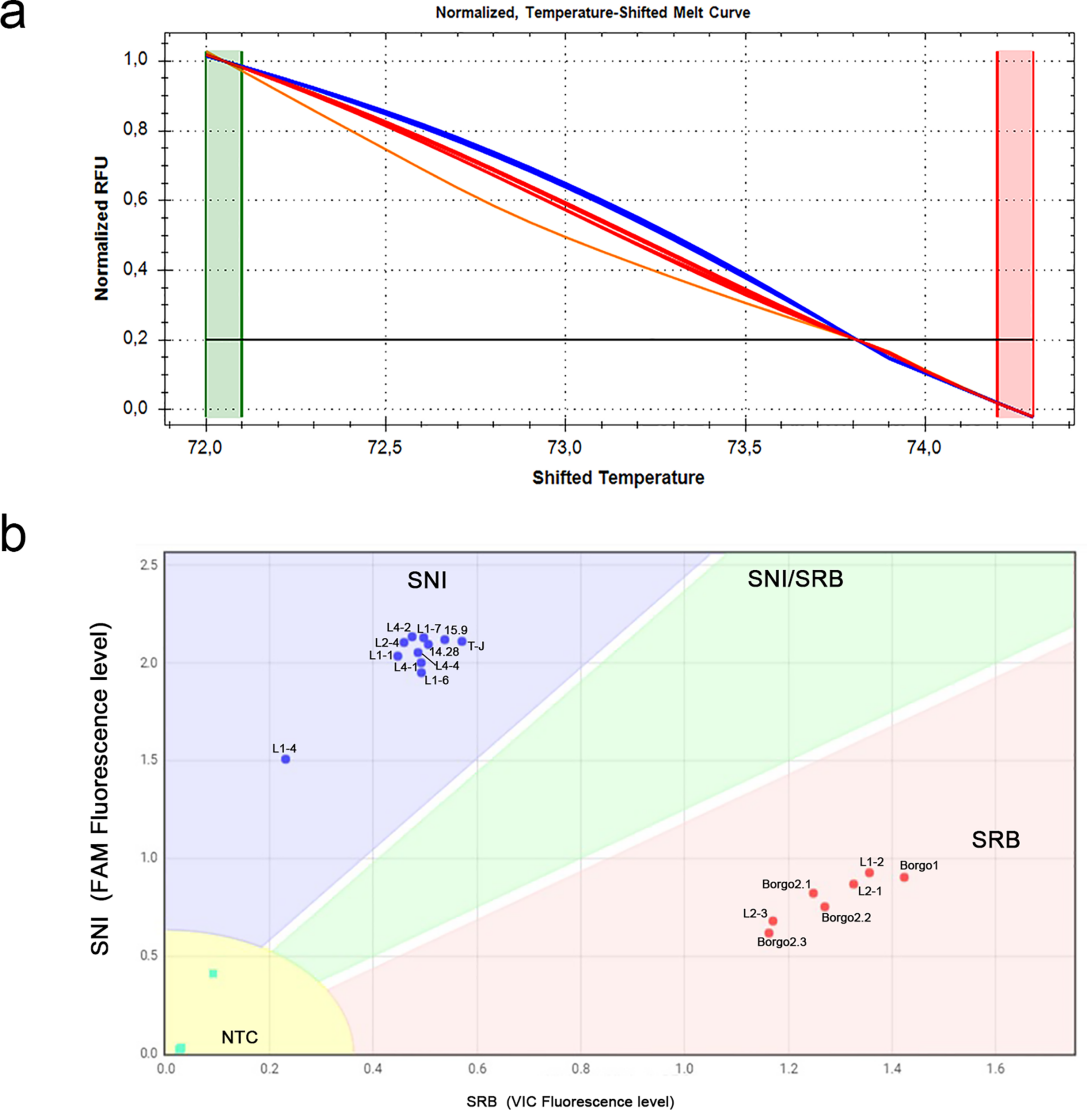
Detection and discrimination of SNI and SRB alleles among different TSWV isolates from tomato and artichoke samples by high resolution melting and custom TaqMan SNP Genotyping assays. (a) The normalized temperature-shifted melt curves relative to HRM analysis assigned with a high percent confidence ranging from 99.8 to 100%, the eighteen samples to three clusters according to reference panel genotypes: SNI (blue), SRB (red) and an additional cluster (orange). (b) The scatter plot from Custom TaqMan SNP Genotyping assays displayed the two biotypes into two boundaries according to the panel reference samples, specifically assigning field isolates to either the SNI allele (within blue boundary) or the SRB allele (red) with a call rate of 100%. Four NTC were included in the analysis.

In HRM analysis the 18 samples, whose cDNA was obtained with gene-specific primer, showed the temperature-shifted difference curve profiles clustered in two major groups. The two clusters consistently identified the biotypes SNI and SRB, except two cases of misclustering consisting either in a SNI allele wrongly called as SRB, or the identification of a further cluster (Fig 4A and Table 1). On the other hand, HRM amplification from cDNA obtained with random primers gave less consistent results, since four samples did not amplify and three were wrongly assigned to an additional cluster (Table 1).

Custom TaqMan SNP assays were more accurate than the HRM assay in discriminating the SNI and SRB alleles. In fact, in the case of RNAs reverse-transcribed with gene-specific primer, all 18 TSWV isolates were successfully amplified and correctly assigned to the SNI or SRB clusters, in complete agreement with Sanger sequencing results when available (Fig 4B and Table 1). When cDNA samples primed by random hexamers were used as the templates, two main clusters corresponding to the SNI and SRB biotypes were identified, but two cases of failure in amplification and one of misclustering were also recorded (Table 1). None of the samples tested from the field collection was identified as bearing a mixed SRB/SNI infection by either SNP assays.

### Identification of SNI and SRB TSWV isolates from tomato crude leaf extracts

**Fig 5:**
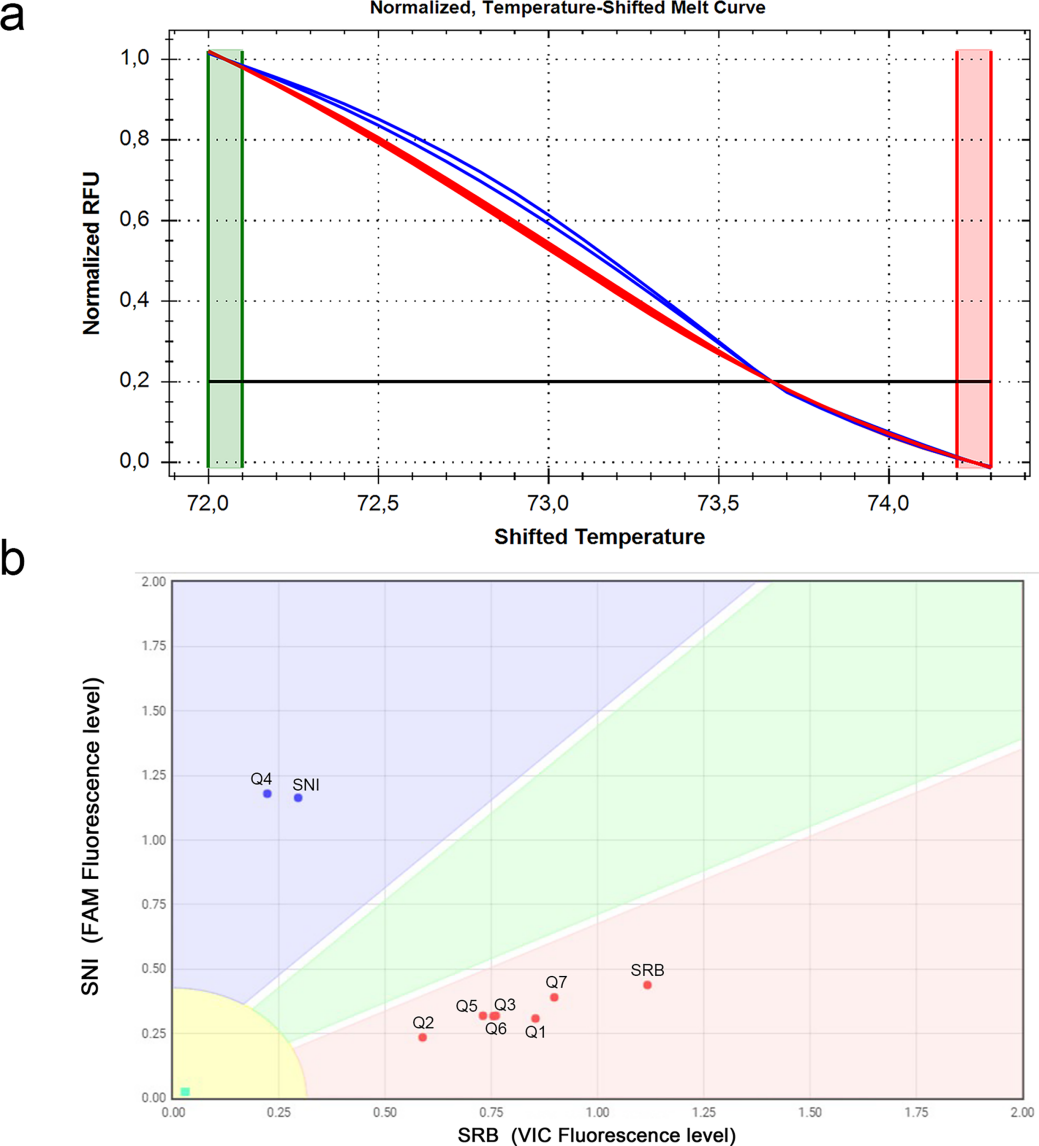
Detection and discrimination of SNI and SRB alleles from crude leaf extracts of tomato plants collected in field conditions by HRM and custom TaqMan SNP Genotyping assays. (a) The temperature shifted melt curves relative to HRM analysis assigned the samples to two clusters according to reference panel genotypes, SNI and SRB, whose colours are associated to reference panel samples like in the previous figures. (b) The scatter plot from Custom TaqMan SNP Genotyping assays identified three boundaries corresponding to SNI, SRB and NTC. All samples were correctly amplified and assigned to the proper biotype.

We tested the ability of the two SNP genotyping techniques to perform reliably when crude leaf extracts (CLEs) were used as the starting material. The CLE method enabled the fast extraction of viral RNA templates from leaf tissues for direct RT-PCR analysis, avoiding grinding samples in liquid nitrogen, using harmful chemicals (i.e. phenol and chloroform) and lengthy precipitation/resuspension procedures. From six non-Sw-5 and one Sw-5 tomato plants, all infected by TSWV in the field, cDNA samples were synthesised from CLEs with the gene-specific primer. The quick CLE method yielded a cDNA template that, upon 1:10 dilution, provided consistent and reproducible results with both the techniques. As shown in Fig 5A and 5B and Table 1, an identical output was achieved for both assays, proving that a quick CLE protocol is suitable for simple (crude leaf extraction, no post-PCR manipulations) and rapid (four hours from plants sampling to diagnostic results) SNP genotyping assay.

## Discussion

Deployment of commercial varieties carrying resistance genes is considered both an economically convenient and an environmentally sound strategy for crop disease management. However, due to the extensive use of such genes in resistant cultivated varieties, the emergence of new virulent strains may occur very rapidly. Viruses' adaptation to resistant cultivars and the fast diffusion of resistance-breaking strains may drastically decrease the applicability of otherwise successful management strategies based on improved genetic resistance [36].

This is the case, for example, of tomato cultivars carrying the dominant gene Sw-5, which confers resistance to TSWV and other Tospoviruses. As soon as Sw-5 was introgressed and widely used in tomato commercial cultivars, appearance of SRB isolates began to represent a high risk for the durability of resistance to TSWV worldwide. Hence, rapid and accurate characterization of TSWV populations for discrimination of SRB biotypes is challenging and pivotal for the tomato spotted wilt disease management.

In this view, our present work provides advice on robust and sensitive diagnostic techniques able to distinguish between TSWV SNI and SRB isolates, that could replace cumbersome and costly practices involving nucleic acid extraction, DNA cloning and sequencing. We developed two PCR-based genotyping assays that could be both implemented as fast and reliable analytical procedures in plant virus diagnostic laboratories. PCR-based methods for detection and genotyping of RNA viruses must include a reverse transcription step for converting the RNA template in the starting cDNA target. Although many real-time RT-qPCR protocols and commercial kits suggest the use of the random priming method in the reverse transcription reaction [37, 38], some authors warn about the possible pros and cons of using random versus target-specific primers for first strand cDNA synthesis [39]. Therefore, our study specifically addressed this methodological feature, proving that the use of a target-specific primer consistently improved the accuracy and the sensitivity of both the downstream PCR-based genotyping assays tested.

Custom TaqMan™ SNP Genotyping Assays provide a powerful and sensitive technology for detection of polymorphisms in a ready-to-use PCR format, and are routinely used for genotyping human disease-related allelic variants [40]. The adoption of the Custom TaqMan™ SNP Genotyping Assays, here applied for the first time to the discrimination of viral sequence variants, streamlined the use of TaqMan fluorescent probes for genotyping of plant viral populations [29, 30]. Additionally, although other authors already used TaqMan probe-based assays for TSWV detection [28, 41–43], in our experiments, the TaqMan assays were able to distinguish between SNI and SRB with the highest specificity, sensitivity and accuracy, providing a valuable tool for TSWV biotype discrimination. TaqMan assays resulted consistent and reliable across experiments when combined to the use of gene-specific reverse primers, and also when a quick CLE protocol was applied for viral RNA reverse-transcription and amplification. In our conditions, the TaqMan assays could reveal a copy number of DNA target as low as 50–70 per reaction, a detection limit substantially lower than previously reported by other authors. In fact, Debreczeni and coworkers [28] indicated 10^5^−10^6^ TSWV M RNA molecules as the lower detection limit of their TaqMan MGB probe assay, whereas Jacquot *et al*. [30] determined a lower threshold of 10^4^−10^5^ PVY RNA molecules in a specific fluorescent TaqMan™ RT-PCR assay.

Although several studies successfully applied HRM to pathogen identification [32–34, 44], in this work it was proved less sensitive than the TaqMan assays. In addition, a long optimization of PCR conditions was needed, involving primer pair design, thermal condition (standard and touchdown) validation, reverse-transcription priming method, and standardization of the starting amount of cDNA template in the PCR reaction. In HRM, the threshold levels for target detection were four orders of magnitude higher than the TaqMan assays. Furthermore, the HRM showed lower specificity and accuracy than the TaqMan assay, since in several cases samples were improperly assigned to the wrong genotype or did not amplify. This might be due to the low quality and concentration of the input material, that likely affects HRM [44] more than TaqMan assays. On the other hand, HRM could be a better option when the detection of mixed infections is sought for. For this latest application, the HRM showed a higher sensitivity than the TaqMan assays in identifying the simultaneous presence of SNI and SRB, especially when they were not equal in concentration. This finding is in agreement with the acknowledged sensitivity of HRM for detecting heterozygotes [45]. Thus, we suggest that once established the protocol, HRM may be used as an alternative to TaqMan SNP assays as a pre-screening technique on large scale monitoring of viral populations in the field, or when infection by mixture of viral isolates is expected. In fact, HRM should be the genotyping approach of choice when investigators aimed to minimize risks of false negative detection of SRB genotypes due to a higher amount of SNI TSWV in mixed infections on non-Sw5 tomato plants or on different crop species.

Although this study is mainly focused on an innovative diagnostic system offered by the two described TSWV genotyping assays, the tests on infected samples from the field suggest some important conclusions from an epidemiological point of view. Indeed, in fields historically affected by SRB TSWV, even non-Sw-5 tomato genotypes mostly harbour the SRB infection. Moreover, as showed, winter-grown crops such as globe artichoke, an alternate crop for tomato in the same growing areas, maintained SRB TSWV in a considerable proportion of infected plants (25%). A recent study suggested that SRB isolates are limited only to Sw-5-carrying tomato in Californian fields, where they could arise from individual mutation events [17]. On the contrary, our study describes a different scenario where SRB TSWV isolates are present year-round, overwintering in alternative crops such as artichoke that function as virus reservoirs. Analysis of a higher number of samples, that is beyond the scope of this work, with one of the SNP discrimination methods here described, could confirm the picture of a stable population of the SRB TSWV biotype established in limited, but expanding, areas of Southern Italy.

In conclusion, the technical advance presented in this work prompts to the following considerations: (i) we develop the first and efficient TaqMan SNP genotyping assays, to address the issue of SNI and SRB TSWV isolates identification, detection and discrimination; (ii) upon a better standardization of experimental conditions, HRM can considerably reduce time and costs of large-scale genotyping studies with a limited risk of failure or misclassification, thus representing a valuable tool for the pre-screening of TSWV populations, and for the simultaneous detection of both SNI and SRB TSWV biotypes; (iii) the herein designed assays enabled sensitive allelic discrimination of TSWV isolates also when applied in rapid protocols including CLE as the template. On the other hand, it is to be considered that at least two polymorphisms have been associated so far to SRB TSWV genotypes [27]. In this study we set up TSWV genotyping assays based on the only SNP known to occur in Italy and other countries [17], but additional work is necessary to confirm that the here presented assays would be applicable with a different set of primers to the other resistance-breaking determining SNP recorded.

These new methods represent valuable tools that, for their high sensitivity and specificity, could be adopted for introducing TSWV biotype discrimination in internationally recognised diagnostic protocols for regulated pests [46]. They could be also flexibly adapted, by using different primers and probes, to any sort of SNP or allelic variant detection found to determine new emerging SRB TSWV genotypes that should occur in the future, or in the case of any other RNA virus species whose monitoring is of crucial interest in disease management programmes.

## Material and methods

### Plant material collection

Leaf samples of tomato (*Solanum lycopersicum*) and globe artichoke (*Cynara scolymus*) were collected at different sites, mainly within an important processing tomato growing area located in the province of Foggia (Apulia, Southern Italy) that suffered severe TSWV outbreaks in recent years. Both Sw-5 and non-Sw-5 commercial tomato hybrids were collected as the infected source material in different fields over two consecutive summers, 2015 and 2016 (all names and geographic coordinates of locations are given in S1 Table and S1 Fig). Artichoke samples from three different hybrids and cultivars were collected in winter 2016 in the same fields that were to host tomato cultivation in the following summer, according to the typical crop rotation in the area of interest (S1 Table). The owners of the fields gave regular permission to conduct the study on their sites and materials. TSWV infection was preliminarily assessed by reverse-transcription PCR (all primers and probes used in this work are listed in Table 3) [26].

**Table 3:** Primers and probes used in the described experimental procedures.

PCR primer name	Sequence 5’-3’		PCR product (bp)	Purpose
TSWV_NSm	For	GAATCAATCAATTACATTACACAAGC	1008	Sanger seq.; Rev. Transcription
	Rev	CAAAATTAACAGAAAYTTAARCTTRRAYAAG		
*HRM-1*	*For*	*GCAAATAAGGTCATCAAG*	*73*	*HRM*
	*Rev*	*TCACAATCCTGGAAATCATC*		
*HRM-2*	*For*	*GCAAATAAGGTCATCAAGATCT*	*47*	*HRM*
	*Rev*	*GTTTTCTGCTGTCCCAAGTTGG*		
HRM-3	For	AAAATGCAAATAAGGTCATCAAG	55	HRM
	Rev	TATTGTTTTCTGCTGTCCCAAGTT		
TSWV_NSm_SNP	For	CGGGAAGCAAAATGCAAATAAGGT	92	TaqMan SNP Assay
	Rev	CCCATATCACAATCCTGGAAATCATCA		
	NSm_SNI_FAM probe	CAAGTTGGACAGATCT		
	NSm_SRB_VIC probe	CCAAGTTGGATAGATCT		

In italics, primers used in the optimization steps.

### RNA extraction and reverse transcription

Total RNA was extracted from approximately 100 mg of green leaf tissue using TRIZOL® Reagent (Thermo Scientific, Waltham, USA) after grinding in liquid nitrogen according to the manufacturer’s instructions. The concentration and quality of RNA was evaluated by a NanoDrop® ND-1000 spectrophotometer (NanoDrop Technologies, Wilmington, USA), and confirmed visually by gel electrophoresis. For the CLE preparation, approximately 100 mg of green leaf tissue were homogenized on ice in a microfuge tube with a conical pestle in the presence of four volumes of extraction solution (0.1% N-lauroyl sarcosine, 0.1% Triton-X100, 40 mM DTT), and solid cellular debris were separated by centrifugation. The supernatant was diluted in 10 mM DTT before reverse transcription. Reverse transcription was performed as follows: after 3 min of denaturation at 95°C, 1 μg of total RNA or 1 μl diluted CLE was converted into cDNA in a 20 μl reaction using the High Capacity cDNA Reverse Transcription Kit (Thermo Scientific), according to the manufacturer’s instructions. The reactions were primed in the presence of either 10 pM random hexamers or the specific reverse primer TSWV_NSm_rev complementary to the region 1024–1054 of the M RNA (Table 3).

### PCR amplification, molecular cloning and DNA sequencing of TSWV NSm gene

Primers TSWV_NSm_For, homologous to position 47–72 of TSWV M RNA, and TSWV_ NSm_Rev (Fig 1) were used to amplify and sequence the NSm gene of several viral isolates. Amplifications were performed in a T100^TM^ thermal cycler (Bio-Rad, Hercules, USA) as described [26]. Amplicons were ligated into the pGEM-T Easy vector (Promega, Madison, USA), and cloned into E. coli DH5α competent cells. DNA from recombinant plasmids were sequenced by the Sanger method by an external sequencing service (Macrogen Europe, Amsterdam, NL). Sequences of the NSm genes of samples 15.9 and Borgo 1 (Table 1) have been deposited (acc. no. MG457157, MG457158).

### Virus copy number calculation and standard curve construction for SNP assays

In order to quantify the TSWV M RNA copy numbers in the total RNA extracts, we performed an absolute quantification with real time PCR on CFX96 Touch™ Real-Time PCR Detection System (Bio-Rad). The viral cDNA standard (sample 15.9) was amplified in a final volume of 50 μl containing 2 μl of cDNA, 1 x Buffer High Fidelity (Thermo Fisher), 0.2 mM dNTPs mix, 0.25 μM of HRM3-For and HRM3-Rev primers, and 1 U of Phusion High-Fidelity DNA polymerase (Thermo Fisher). The amplification protocol was set as follows: an initial denaturation at 98°C for 30 s, 35 cycles at 98°C for 10 s, 60°C for 15 s, 72°C for 10 s and a final extension at 72°C for 5 min. Then, the PCR specificity was evaluated on 3% agarose gel electophoresis and the bands corresponding in size and molecular weight to the expected products were gel-excised and purified by using QIAEX II gel extraction kit (QIAGEN, Hilden, Germany), following manufacturer’s instructions. Afterwards, to calculate the copy number of the amplicons in each reaction, the purified gene fragments were quantified using NanoDrop® ND-1000. The number of molecules in the standard was determined [47]. Four serial decimal dilutions of the standard and 3 dilutions of the cDNA samples of unknown concentration (15.9 and Borgo 1) were used for standard curve construction. Absolute quantification using real time PCR was performed in triplicate, along with a NTC, in a final volume of 10 μl using 1.5 μl of template, 0.4 μM for each primer, and 1 x SYBR™ Green Master Mix for CFX (Applied Biosystems, Foster City, USA). The thermal cycle protocol was set up using the following conditions: enzyme activation at 50°C for 2 min, an initial denaturation at 95°C for 2 min, 40 cycles composed of denaturation at 95°C for 15 s and one step annealing/extension at 60°C for 1 min, with fluorescence signal acquisition at the end of each cycle. The amplification protocol was followed by the melt curve step starting from 65°C to 95°C with and increment of 0.5°C every 5 s, with plate fluorescence read each cycle. Thereafter, amplification plot, quantification data and melt peak were analysed using the software CFX Manager v 3.1 (Bio-Rad).

### High-resolution melting analysis

High resolution melting analysis was performed on CFX96 Touch™ Real-Time PCR Detection System (Bio-Rad). Three primer pairs, surrounding the SNP mutation discriminating between SNI and SRB genotypes (Fig 1 and Table 3), were tested to establish the optimal PCR efficiency. HRM analyses were carried out in a final volume of 20 μl, consisting of 1:5 diluted cDNA (1 μl), 1 x SsoFast™ EvaGreen® Supermix (Bio-Rad), and 0.4 μM forward and reverse primers. A NTC was included in each run. Four cycling programs were tested, consisting of a standard protocol as suggested by the manufacturer, and three touchdown protocols [48] modified by starting from three annealing temperature: 57, 60 and 63°C. After verification of robust amplification curves, the melting curve stage was further analysed by Precision Melt Analysis™ Software (Bio-Rad), which automatically elaborates the melt file. Some parameters were manually adjusted to increase the stringency used to classify melt curves into different clusters. In particular, the pre-melt and post-melt range was set up from 72.0°C to 72.1°C, and from 74.2°C to 74.3°C, respectively. Temperature-shift bar height was set up at 0.20°C and the melt curve shape sensitivity was fixed at 25 to yield more homozygous clusters. The melting temperature difference threshold (Tm), which determines the lowest amount of Tm difference between samples through which the software will call as different cluster, was set at 0.25°C for cluster detection.

### Custom TaqMan® SNP Genotyping assays

To amplify and detect the specific single nucleotide polymorphism (A>G transition) in cDNA samples deriving from either SNI or SRB TSWV genotypes, or from *in vitro*-assembled mixtures of the two, we adopted a Custom TaqMan® SNP Genotyping Assays (Thermo Scientific). The genotyping assay was performed in a StepOnePlus™ Real-Time PCR System (Applied Biosystems) in a reaction volume of 12.5 μl consisting of 1 x Taqman® Genotyping Master Mix, 1 x Taqman® SNP Genotyping Assay (containing primers and probes indicated in Table 3) and 1μl of 1:5 diluted cDNA samples. As suggested by the manufacturer’s protocol, at least two NTC were included in the assay in order to orient the VIC® and FAM™-dye clusters to an origin and to evaluate the presence of contamination. Furthermore, SNI and SRB positive controls were included. The Genotyping™ Software freely available on the web was used for data analysis (https://www.thermofisher.com/it/en/home/cloud.html). The software analysis options were set choosing "classification scheme" as the call method and "normalised clusters", to minimise run to run variations for maximal overlap of genotyping clusters across experiments.

## Supporting information

S1 Table Details on the plant samples used in this work. Information on isolate name, site of sampling with geographic coordinates, plant species, resistance to TSWV, genotype and seed company is given.
(DOCX)

S1 Fig Map of four sites in the Foggia province (Southern Italy) where TSWV isolates used in this study were collected. (DOCX)
