# Supplementary material for: Rapid identification of tomato Sw-5 resistance-breaking isolates of Tomato spotted wilt virus using high resolution melting and TaqMan SNP Genotyping assays as allelic discrimination techniques
Source: PLoS One. 2018 Apr 30;13(4):e0196738. doi: 10.1371/journal.pone.0196738 (PMC5927427; doi:10.1371/journal.pone.0196738)
Supplement: S1 Table — Information on isolate name, site of sampling with geographic coordinates, plant species, resistance to TSWV, genotype and seed company is given. (DOCX) [file pone.0196738.s001.docx]

Supporting Information

TABLES

Table S1. Details on the plant samples used in this work. Isolate name, site of sampling including geographical coordinates (DMS system), plant species, resistance to TSWV, genotype and company

| **NO.** | **ISOLATE** | **SITE *** | **CROP** | **TSWV resistance** | **Genotype** | **Company** |
| --- | --- | --- | --- | --- | --- | --- |
| 1 | Borgo 1 | Cerignola^1^ | Tomato | Sw-5 | Impact F1 | ISI |
| 2 | Borgo 2.1 | Borgo Libertà^2^ | Tomato | Sw-5 | Docet F1 | Seminis |
| 3 | Borgo 2.2 |  | Tomato | Sw-5 | Taylor F1 | Nunhems |
| 4 | Borgo 2.3 |  | Tomato | Sw-5 | Discovery F1 | ISI |
| 5 | T-J |  | Tomato | - | Jag F1 | Seminis |
| 6 | 15.9 | Fasano^3^ | Tomato | - | Nemanaxos F1 | Nunhems |
| 7 | Q1 | Stornara^4^ | Tomato | - | Talent | Esasem |
| 8 | Q2 |  | Tomato | - | Talent | Esasem |
| 9 | Q3 |  | Tomato | - | Talent | Esasem |
| 10 | Q4 |  | Tomato | - | Talent | Esasem |
| 11 | Q5 |  | Tomato | - | Talent | Esasem |
| 12 | Q6 |  | Tomato | - | Talent | Esasem |
| 13 | Q7 |  | Tomato | Sw-5 | Docet F1 | Seminis |
| 14 | 14.28 | Fasano^3^ | Artichoke | - | Opal F1 | Nunhems |
| 15 | L1-1 | Orta Nova 1^5^ | Artichoke | - | Opal F1 | Nunhems |
| 16 | L1-2 |  | Artichoke | - | Opal F1 | Nunhems |
| 17 | L1-4 |  | Artichoke | - | Opal F1 | Nunhems |
| 18 | L1-6 |  | Artichoke | - | Opal F1 | Nunhems |
| 19 | L1-7 |  | Artichoke | - | Opal F1 | Nunhems |
| 20 | L2-1 | Orta Nova 2^6^ | Artichoke | - | Madrigal F1 | Nunhems |
| 21 | L2-3 |  | Artichoke | - | Madrigal F1 | Nunhems |
| 22 | L2-4 |  | Artichoke | - | Madrigal F1 | Nunhems |
| 23 | L4-1 | Orta Nova 4^7^ | Artichoke | - | Spinoso Sardo |  |
| 24 | L4-2 |  | Artichoke | - | Spinoso Sardo |  |
| 25 | L4-4 |  | Artichoke | - | Spinoso Sardo |  |

* All sampling sites are located in the Foggia province (see Fig. S1), except Fasano that is located in the Brindisi province, about 170 km afar in South -East direction.

^1^ Cerignola (FG) - Italy (DMS: N 41° 14' 43.822'', E 15° 49' 56.913'')

^2^ Borgo Libertà, Cerignola (FG) - Italy (DMS: N 41° 10' 9.546'', E 15° 40' 58.74'')

^3^ Fasano (BR) - Italy (DMS: N 40° 52' 5.03'', E 17° 22' 23.008'')

^4^ Stornara (FG) - Italy (DMS: N 41° 18' 11.2'', E 15° 45' 6.199'')

^5^ Orta Nova (FG) - Italy (DMS: N 41° 20' 60'' E 15° 45' 30'')

^6^ Orta Nova (FG) - Italy (DMS: N 41° 21' 6.03'', E 15° 45' 37'')

^7^ Orta Nova (FG) - Italy (DMS: N 41° 21' 1'', E 15° 45' 33'')
