# Supplementary material for: Rapid identification of tomato Sw-5 resistance-breaking isolates of Tomato spotted wilt virus using high resolution melting and TaqMan SNP Genotyping assays as allelic discrimination techniques
Source: PLoS One. 2018 Apr 30;13(4):e0196738. doi: 10.1371/journal.pone.0196738 (PMC5927427; doi:10.1371/journal.pone.0196738)
Supplement: S1 Fig — (DOCX) [file pone.0196738.s002.docx]

Supporting Information

FIGURE S1


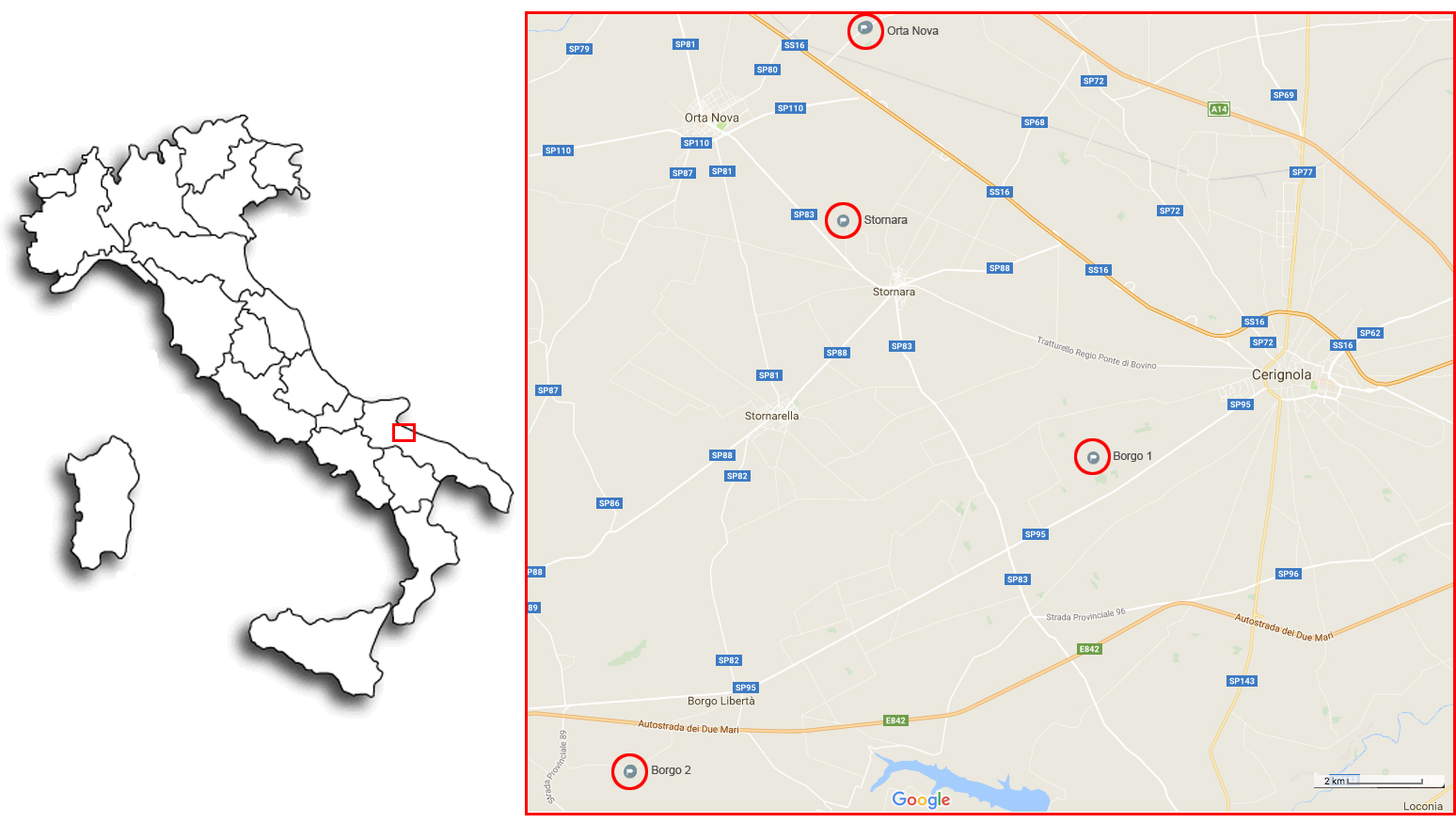


**Fig. S1** Map of four sites in the Foggia province (Southern Italy) where TSWV isolates used in this study were collected.
